# *de novo* interstitial deletions at the 11q23.3-q24.2 region

**DOI:** 10.1186/s13039-016-0247-7

**Published:** 2016-05-05

**Authors:** Jiasun Su, Rongyu Chen, Jingsi Luo, Xin Fan, Chunyun Fu, Jin Wang, Sheng He, Xuyun Hu, ShuJie Zhang, Shang Yi, Shaoke Chen, Yiping Shen

**Affiliations:** Department of Genetic and Metabolic Central Laboratory, Guangxi Maternal and Child Health Hospital, No59 Xiangzhu Road, Nanning, China; Department of Laboratory Medicine, Boston Children’s Hospital, 300 Longwood Avenue, Boston, MA 02115 USA

**Keywords:** Jacobsen syndrome, Contiguous gene deletion syndrome, Chromosomal microarray, Interstitial deletion

## Abstract

**Background:**

Jacobsen syndrome (JBS) is a contiguous gene deletion syndrome involving 11q terminal deletion. Interstitial deletions at distal 11q are rare and their contributions to the clinical phenotype of JBS are unknown.

**Case presentation:**

We presented the chromosome microarray (CMA) data and the clinical features of two individuals carrying a non-overlapping *de novo* deletion each at the 11q23.3-q24.2 region in an effort to analyze the correlation between region of deletion at 11q and phenotype. Both deletions are likely pathogenic for patient’s condition. The deletion at 11q23.3q24.1 is associated with short stature, relative microcephaly, failure to thrive, hypotonia and sleeping disorder. The deletion at 11q24.2 involves *HEPACAM* and our patient’s clinical presentation (relative macrocephaly, abnormal MRI, mild developmental delay and seizure) is not inconsistent with Megalencephalic leukoencephalopathy with subcortical cysts 2B.

**Conclusions:**

Our finds support the notion that more than one critical region at 11q23.3-qter are responsible for the variable clinical presentation of JBS, thus JBS is a true contiguous gene deletion syndrome where multiple loci contributed to the clinical characteristics of JBS. Small interstitial deletions at 11q23.3-q24.2 and their associated unique features also suggest emerging novel genomic disorders.

**Electronic supplementary material:**

The online version of this article (doi:10.1186/s13039-016-0247-7) contains supplementary material, which is available to authorized users.

## Background

The 11q terminal deletion, also known as Jacobsen syndrome (OMIM #147791), is a contiguous gene deletion syndrome involving the deletion of 11qter. Several hundred 11q terminal deletion patients had been described since it was first reported by Jacobsen in 1973 [1]. The deletions observed in JBS patients range from 7 to 20 Mb in size and are almost always associated with 11q terminal deletions and the furthest breakpoint is located at 11q23.3. Typical features of JBS include developmental delay (DD)/intellectual disability (ID), dysmorphic facial features, platelet disorder and multiple congenital defects [1–6]. Interstitial deletions from 11q23 to 11qter are rare and their clinical significance is currently unknown. Some of previously reported 11q23-qter interstitial deletions were characterized by karyotyping analysis [7–9]. CMA analysis made it possible to better define the genotype–phenotype correlation. Since the first report of an 11q24.1q24.3 interstitial deletion characterized by CMA [10], eight cases with 11q23-qter interstitial deletion detected by CMA had been documented in literature (see Additional file 1: Table S1). The breakpoints of the 11q23-qter interstitial deletions are variable and deletions range from 2.89 to 12.8 Mb in size. Clinical presentations of affected individuals are also very heterogeneous, thus complicating the genotype–phenotype correlation analysis [4, 5, 10–14]. Patient with interstitial deletion at 11q23-qter region could share some of the JBS features such as hypotonia [9], macrocephaly [11, 14], microcephaly [5], trigonocephaly [4, 7, 9], some of dysmorphic facial features [4, 5, 7–14], limbs anomalies [7, 11, 13], congenital heart defect (CHD) [4, 5, 7, 9, 10, 12], but often did not exhibit the whole constellation of JBS [13, 14]. The most consistent feature among all patients with 11q23-qter interstitial deletion is DD/ID. Smallest overlapping regions helped to define some critical regions or candidate genes at the distal 11q region [4–11].

Here, we reported two individuals carrying non-overlapping interstitial deletions at 11q23.3-q24.2 region with different degrees of DD/ID and some dysmorphic features in an effort to further define genotype–phenotype correlation.

## Case presentation

### Patient 1

Patient 1 (P1) was a 5-year-old girl born to a 29-year-old woman at 41 weeks of gestational age. Pregnancy and delivery were uneventful. Her birth weight was 2.8 kg (~15^th^percentile) and birth length around 48.2 cm (~15^th^percentile). She had an apparently healthy twin brother and sister. At around 5–6 months of age she was noticed to have global delay including unable to roll over, delayed crawling, sitting and standing. She was at a 5-month developmental level at the age of one. She had a history of failure to thrive and hypotonia. She came to Genetics Clinic due to dysmorphic features and global developmental delay. At 5-years of age, her height was 101 cm (−2.11 SD), weight 14 kg (−2.58 SD) and head circumference 48.3 cm (7th percentile). She had overlapping toes, dysmorphic features including mild hypertelorism (inner canthal distance 27 mm, +1SD), prominent forehead, flat facial profile, broad nose, smooth philtrum with thin upper lip, upslanting palpebral fissures with epicanthal folds. Her brain MRI was normal. No other defect was noticed.

She had a history of sleeping disorder. Notably, her parents reported that she would vocalize around bedtime, often it is associated with body rocking and head banging. She would wake up and bang her head at midnight. She would sit Indian style on her bed and leaned forward until she banged her head on the mattress. This occurred in a fairly fast and rhythmic pattern. Often she would moan during such a movement and many times the moan escalated into very loud scream. She also had the rocking behavior during the daytime that she would move her back up against a hard surface and then rocked her lower back and buttocks on the floor. She was diagnosed as jactatio capitis nocturna and such behavior had improved but not resolved entirely.

### Patient 2

Patient 2 (P2) was a boy who came to hospital due to fever, mild developmental delay and seizure at the age of 10 months. He was a full term second child to a healthy parent who had a healthy 4-year-elder daughter. He was delivered via cesarean section without complication during pregnancy or delivery. His birth weight was 3.25 kg (25–50th percentile) and birth length was 50 cm (25–50th percentile). He had no significant medical history prior to presentation. No family history of seizure, autism, mental retardation or other neurologic impairments. He raised his head around 3–4 months of age, rolled over around 6–7 months of age, and pronounced first word at about 10 months of age. His developmental quotient (DQ) and the mental index (MI) suggested that he had mild development delay by the Denver Development Screening Test score (DQ = 51 and MI = 63). At the age of 10 months, his weight was 9.4 kg (30th percentile) and height 72.5 cm (25th percentile). He presented with a relative macrocephaly (circumference was 47.5 cm (+1.3SD)), mild hypertelorism, low nasal bridge, thin upper lip and strabismus. The magnetic resonance imaging (MRI) revealed the widening of the frontotemporal lobe, full bilateral ventricles and deep parietal sulci. He had normal electroencephalogram and electromyography. He was diagnosed with acute pharyngitis, secondary seizure, psychomotor retardation and mild development delay as well as cerebral hypogenesis.

## Method

### Chromosomal microarray (CMA) analyses

DNA sample was extracted from peripheral blood lymphocytes using standard protocol, microarray was performed using Agilent 244 K array for patient 1 and the illumina HumanSNP cyto-12 array for patient 2.

## Results

**Fig. 1:**
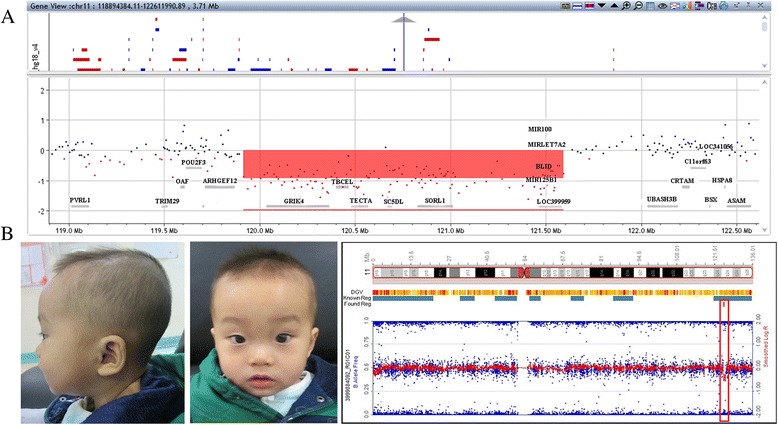
**a** The deletion (shaded in red) at 11q23.3-q24.1 in P1 detected by Agilent 4X180K array. **b** The facial features of P2. Large forehead, mild hypertelorism, low nasal bridge, thin upper lip and strabismus. The illumina array detected a small deletion (*red rectangle*) at 11q24.2

A 1.6 Mb deletion at the region of 11q23.3-q24.1 (chr11:120410050–122085906) (hg19) was detected in the genome of P1. A 0.76 Mb deletion at 11q24.2 (chr11:124635144–125390604) (hg19) was detected in the genome of P2 (Fig. 1). Parental CMA demonstrated that both deletions were *de novo*.

## Discussion

Interstitial deletions on 11q are rare and the genotype-phenotype study had been hindered by the small number of cases reported and a lack of sufficient details about patient’s clinical phenotype. So far there are only 11 interstitial deletion cases at the 11q23.3-q25 region have been reported (Additional file 1: Table S1). Chromosome microarray data were available for eight of them. Guerin et al. reported a patient with a 2.899 Mb interstitial deletion at 11q24.2-q24.3, presented with trigonocephaly, hypertelorism and deep-set eyes [4]; Taoyun Ji et al. reported a patient with a 4.11 Mb interstitial deletion at 11q25, presented with severe DD, microcephaly and some facial dysmorphism [5]; Tyson et al. described a patient with a 4.74 Mb deletion at 11q24.2q24.3, presented with macrocephaly, ID and abnormal MRI [11]; SO J et al. reported a woman with a 3.162 Mb interstitial deletion at 11q24.2-q24.3, presented with periventricular nodular heterotopia and transverse limb reduction defect [13]; Yamamoto et al. identified a 20-month-old boy with a 5.3 Mb interstitial deletion at 11q23.3q24.2 and presented with white matter abnormalities, prenatal macrocephaly and mild developmental delay [14]. Two patients reported here added to this list. P1 presented with short stature, DD/ID, relative microcephaly, sleeping disorder, some dysmorphic features and P2 exhibited mild DD/ID, relative macrocephaly, mild hypertelorism. A summary of the clinical features of the eight previously reported patients with 11q23-qter interstitial deletions and two present cases is listed in Table 1. The prevalence of phenotype among these interstitial deletion patients can not be quantified for the small number, but the summarization collectively support the notion that Jacobsen syndrome is a contiguous gene deletion syndrome where multiple genes (regions) involved within the deletion region play roles to the syndromic phenotypes.

**Table 1:** Summary of clinical features of the eight patients with 11q23-qter interstitial deletions and our present cases

Clinical Findings	Previously cases	P1	P2
Number	8	1	1
Region	11q23-q25	11q23.3q24.	11q24.2
Deletion size (Mb)	2.89–12.8	1.6	0.76
Gender	3 m/5f	f	m
Age	Ranges from newborn to adult	4 years	10 months
Birth weight	Low-normal	~15thpercentile	25–50th percentile
Hypotonia	-	+	-
Macrocephaly	2	-	relative macrocephaly
Microcephaly	2	relative microcephaly	-
Trigonocephaly	1	-	-
Prominent forehead	1	+	-
Hypertelorism	2	mild	mild
Palpebral fissure anomalies	4	+	-
Ear anomalies	4	-	-
Nasal anomalies	3	+	+
Mouth anomalies	3	+	+
Limb anomalies	2	+	-
Cardiovascular anomalies	4	-	-
Hematological anomalies	3	-	-
Developmental delay/intellectual disability	6	+	+
Social interaction difficulties	2	-	NA

Clinical findings: +, present; −, absent, *f* female, *m* male, *NA* not applicable

**Fig. 2:**
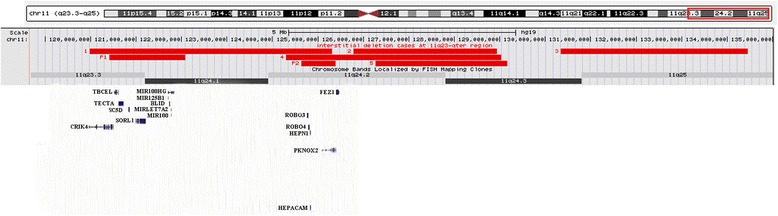
Schematic representation referred to interstitial deletion at 11q23-qter region cases reported (UCSC Genome Browser, hg19)

DD/ID is a consistent feature of JBS. It is also a common feature of patients with 11q interstitial deletions (Table 1). Taoyun Ji et al. proposed a critical region for DD/ID near the telomere defined by a 4.1 Mb deletion (case 3 in Fig. 2) [5]. The deletion detected in our P2 is the smallest in size, it overlaps with deletions detected in case 1 [13] and 4 [11]. All three cases had the clinical presentation of DD/ID, based on this observation, we propose a novel DD/ID locus at 11q24.2 (chr11:124635144–125390604). Similarly, the deletion detected in our P1 may define another novel DD/ID locus at 11q23.3-q24.1 (chr11: 120410050–122085906). Thus there are multiple loci on 11q that the haploinsufficiency of each region is likely associated with DD/ID.

It is interesting to note that patient 1 exhibited the characteristic features of jactatio capitis nocturna, also known as rhythmic movement disorder (RMD). Most of RMD will spontaneously resolve by 4 years of age [15]. Our patient’s condition had improved but not resolved entirely at age 5. The underlying cause of RMD is currently unknown, the deletion in P1 may provide a clue for investigating the molecular mechanism of RMD. 13 RefSeq genes including 10 OMIM genes (*GRIK4*, *LRRC35*, *TECTA*, *SC5DL*, *SORL1*, *MIR100HG*, *MIR125B1*, *BLID*, *MIRLET7A2* and *MIR100*) are involved in the deletion. But it is unclear which gene or genes are likely responsible for the sleeping disorder. *GRIK4* (OMIM #600282) encodes a protein that belongs to the glutamate-gated ionic channel family. Glutamate functions as the major excitatory neurotransmitter in the central nervous system through activation of ligand-gated ion channels and G protein-coupled membrane receptors. Takenouchi T et al. and Pickard BS et al. suggested that the haploinsufficiency of *GRIK4* was related to DD, mental retardation, schizophrenia and bipolar disorder [16, 17]. The developmental delay present in our patient may be explained by the *GRIK4* deletion.

The deletion interval in P2 encompassed 6 OMIM genes (*ROBO3, ROBO4, HEPACAM, HEPN1, PKNOX2* and *FEZ1*). Sequence variants in *HEPACAM* (OMIM #611642) have been shown to cause Megalencephalic leukoencephalopathy with subcortical cysts 2A (an autosomal recessive form MLC2A, OMIM #613925) and 2B (an autosomal dominant form MLC2B, OMIM #613926), both of which are characterized with macrocephaly, abnormal MRI and variable degree of intellectual disability [18–20]. The clinical presentation of the autosomal form is milder, some features improve with age. Recently haploinsufficiency of *HEPACAM* was considered as a cause of two patients with heterozygous deletion at 11q23.3q24.2 interstitial deletion 11q24 and clinical features of MLC [14]. P2 was presented with relative macrocephaly, abnormal MRI mild developmental delay and seizure, which is not inconsistent with MLC2B. The much smaller deletion detected in P2 overlap with the interstitial deletion in patient 2 of Yamamoto’s report. The smallest overlapping region between this two cases can exclude the involvement of *FEZ1* (OMIM #604825) gene which plays a role in axonal outgrowth and has been proposed as a candidate gene for abnormal MRI by Tyson et al. [11].

## Conclusions

In summary, we described two rare interstitial *de novo* deletions in JBS region. ID/DD is a shared feature with JBS, supporting the notion that Jacobsen syndrome is a true contiguous gene deletion syndrome and critical regions of ID/DD exist in different regions of 11q terminal. Our study further defined a smallest critical region associate with DD/ID. Each interstitial deletion also presented with its unique features and suggested distinct novel genomic imbalance disorder.

### Consent

Written informed consent was obtained from the parents of the proband for publication of this Case Report and any accompanying images. The consent form was approved by the ethical committee of Guangxi Maternal and Child Health Hospital, China. A copy of the written consent is available for review by the editor of this journal.

## Additional file

Additional file 1: Clinical features from the literature of patients with 11q23-qter interstitial deletions. (DOCX 20 kb)
