# Supplementary material for: de novo interstitial deletions at the 11q23.3-q24.2 region
Source: Mol Cytogenet. 2016 May 5;9:39. doi: 10.1186/s13039-016-0247-7 (PMC4858824; doi:10.1186/s13039-016-0247-7)
Supplement: Additional file 1: — Clinical features from the literature of patients with 11q23-qter interstitial deletions. (DOCX 20 kb) [file 13039_2016_247_MOESM1_ESM.docx]

**Supplement**

**Table S1: Clinical features from the literature of patients with 11q23-qter interstitial deletions**

|  | **SIROTAF et al. [1984]** | **Ono et al.[1996]** | **Pivnick et al. [1996]** | **Wenger et al. [2006]** | **Tyson et al. [2008]** | **Laura et al. [2009]** | **Taoyun Ji et al. [2010]** | **Taoyun Ji et al. [2010]** | **Guerin et al. [2012]** | **So J et al. [2014]** | **Yamamoto et al.[2015]** |
| --- | --- | --- | --- | --- | --- | --- | --- | --- | --- | --- | --- |
| **deleted region** | *11q23.2q25* | *11q21q23.3* | *11q23q25* | *q24.1q24.3* | *11q24.2q24.3* | *11q24.2q25* | *11q25* | *11q23q25* | *11q24.2q24.3* | *11q24.2q24.3* | *11q23.3q24.2* |
| **position** |  |  |  | **120.9-130.7Mb** | **124295999**  **-129036227** | **124453493-132625777** | **130334608**  **-134449983** | **119981839**  **-132808685** | **126272880**  **-129172341** | **125780310-128942331** | **119,982,356-125310469** |
| **detected methods** | ***Karyotype*** | ***Karyotype*** | ***Karyotype*** | ***microarray*** | ***Array CGH*** | ***snp array*** | ***SNP array*** | ***SNP array*** | ***Array CGH*** | ***CGHmicroarray*** | ***CGH microarray*** |
| **size (Mb)** |  |  |  | **10** | **4.74** | **8.2** | **4.1** | **12.8** | **2.89** | **3.16** | **5.3** |
| **gender** | **female** | **female** | **female** | **female** | **male** | **female** | **male** | **female** | **female** | **female** | **male** |
| **gestation(weeks)** |  |  | **term** | **34** | **term** | **41** |  |  | **term** |  | **41** |
| **Brith weight(g)** | **3000** |  | **2165** |  | **25th percentile** | **-2SD** | **low** |  | **3800** |  | **3105** |
| **birth length(cm)** | **53** |  |  |  |  |  |  |  |  |  | **49.4** |
| **age** | **2.5** | **3** | **0.9** | **2** | **7** | **1** | **0.6** | **2.5** | **4** | **67** | **0.2** |
| **hypotonia** | **-** | **-** | **+** | **-** | **-** | **-** | **-** | **-** | **-** | **-** | **-** |
| **macrocephaly** | **-** | **-** | **-** | **-** | **+** | **-** | **-** | **-** | **-** | **-** | **+** |
| **Microcephaly** | **-** | **-** | **-** | **-** | **-** | **-** | **+** | **+** | **-** | **-** | **-** |
| **trigonocephaly** | **+** | **-** | **+** | **-** | **-** | **-** | **-** | **-** | **+** | **-** | **-** |
| **prominent forehead** | **-** | **-** | **+** | **-** | **+** | **-** | **-** | **-** | **-** | **-** | **-** |
| **hypertelorism** | **+** | **+** | **+** | **+** | **-** | **-** | **-** | **-** | **+** | **-** | **-** |
| **slanting of palpebral fissures** | **+** | **-** | **+** | **+** | **-** | **+** | **-** | **-** | **-** | **ptosis** | **left ptosis** |
| **Ear anomalies** | **+** | **-** | **+** | **+** | **+** | **+** | **-** | **-** | **-** | **+** | **-** |
| **Nasal anomalies** | **+** | **-** | **+** | **-** | **+** | **+** | **-** | **-** | **+** | **-** | **-** |
| **Mouth anomalies** | **+** | **+** | **-** | **+** | **-** | **+** | **-** | **-** | **-** | **+** | **-** |
| **Short neck** | **-** | **+** | **-** | **-** | **-** | **+** | **-** | **-** | **-** | **-** | **-** |
| **Limbs anomalies** | **+** | **-** | **-** | **-** | **+** | **-** | **-** | **-** | **-** | **+** | **-** |
| **Cardiovascular** | **CHD** | **N** | **AN** | **AN** | **-** | **VSD** | **N** | **CHD** | **VSD** | **-** | **-** |
| **Hematological anomalies** | **-** | **-** | **-** | **+** | **-** | **+** | **-** | **-** | **+** | **-** | **-** |
| **neurological** | **-** | **seizures, DD** | **MRI abnormal DD** | **DD** | **abnormal MRI DD/ID** | **-** | **DD** | **DD** | **ASD** | **N** | **mild DD** |
| **genitourinary anomalies** | **+** | **-** | **-** | **-** | **-** | **-** | **-** | **-** | **-** | **-** | **+** |
| **others** |  |  |  |  | **difficulties in social interaction** |  |  |  | **difficulties in social interaction** |  |  |

+,present;-,absent;N,normal;AN,abnormal
